# Quantitative methods for genome-scale analysis of *in situ *hybridization and correlation with microarray data

**DOI:** 10.1186/gb-2008-9-1-r23

**Published:** 2008-01-30

**Authors:** Chang-Kyu Lee, Susan M Sunkin, Chihchau Kuan, Carol L Thompson, Sayan Pathak, Lydia Ng, Chris Lau, Shanna Fischer, Marty Mortrud, Cliff Slaughterbeck, Allan Jones, Ed Lein, Michael Hawrylycz

**Affiliations:** 1 Allen Institute for Brain Science, N. 34th Street, Seattle, WA 98103, USA

## Abstract

With the emergence of genome-wide colorimetric *in situ *hybridization (ISH) data sets such as the Allen Brain Atlas, it is important to understand the relationship between this gene expression modality and those derived from more quantitative based technologies. This study introduces a novel method for standardized relative quantification of colorimetric ISH signal that enables a large-scale cross-platform expression level comparison of ISH with two publicly available microarray brain data sources.

## Abstract 2

This study introduces a novel method for standardized relative quantification of colorimetric in situ hybridization signal that enables a large-scale cross-platform expression level comparison of in situ hybridization with two publicly available microarray brain data sources.

## Background

The analysis and interpretation of gene expression data from diverse expression profiling projects present formidable challenges. From a technology perspective, all gene expression profiling methods seek to measure some function of mRNA abundance, and the available platforms include RT-PCR, oligonucleotide and cDNA microarrays, and serial analysis of gene expression (SAGE) [1] and its variants. The recent availability of genomic scale colorimetric *in situ *hybridization (ISH) data [2] adds still another data modality to the mix, one for which strict quantification is more limited and comparison with existing gene expression data is challenging. To properly interpret data sets such as the Allen Brain Atlas (ABA) [3], it is essential to understand the utility and limits of non-radioactive colorimetric ISH signal and to determine the feasibility of comparing this data modality to the dominant gene expression platforms. This study undertakes this goal, and after a technique for relative signal measurement of colorimetric ISH data is presented, the ABA ISH data [2] are compared with publicly available expression data from two microarray sources [4,5] in six major structures of the C57Bl/6J adult mouse brain.

ISH involves anatomic localization of labeled RNA or DNA probes that hybridize to target complementary RNA or DNA sequences in the cell. These hybrids are detected either by using an isotopic probe and emulsion autoradiography, or by non-isotopic methods using specific antibodies to detect a hapten incorporated into the probe [6]. One variation of this methodology involves using dual ISH to detect the expression of two different mRNAs within the same brain section, allowing for the identification of transcripts co-localized in the same cell [7]. Both radioactive and non-isotopic ISH have become powerful tools for detecting and localizing mRNA, particularly in complex tissues with non-uniform structure such as the brain [8,9]. Advantages of radioisotopic ISH are perceived to be sensitivity, quantitative labeling, and relatively unambiguous discrimination of signal versus background [10], although there is evidence that proper use of certain digoxigenin non-radioactive methods are equally sensitive [11]. While being prone to confusing non-specific hybridization products with low level expression [12], non-radioactive colorimetric ISH methods, on the other hand, often provide better anatomic localization and discrimination between cells [13,14], as well as provide a measure of density of labeled cells, a quality lacking with other quantitative assays. Although non-isotopic ISH is generally considered less quantitative [15], to date there have been no systematic efforts to compare genome-scale expression profiles with more traditional quantitative methods.

One characteristic advantage of colorimetric ISH over its radioactive counterpart is its high throughput suitability, an essential component of large-scale studies. The ABA is a genomic scale ISH project that has generated a cellular level resolution gene expression atlas of the adult C57BL/6J brain. The ABA expression profiles are based on a high-throughput colorimetric ISH technique [2,16] in which digoxigenin-labeled riboprobes specific to each gene are hybridized to cellular mRNA transcripts in brain tissue sections. Following tyramide signal amplification to maximize sensitivity, colorimetric detection of the bound riboprobe produces a vivid blue/purple label in cells expressing a particular gene. Image capture is accomplished via an automated microscopy platform, and a suite of automated informatics algorithms has been developed to anatomically map and measure signal expression levels and cellular densities from individual tissue sections and across neuroanatomic regions [2,17]. These methods provide genome-wide, region-specific quantification of ISH signal that can be directly compared with available microarray data sets.

In comparison with other techniques for measuring gene expression, ISH data quantification presents particular challenges. Signal quantification methodologies for radioactive ISH based on optical density and light transmittance through a tissue have been rigorously established [15,18,19]. By contrast, it is generally held that there are few options for exact quantitative measurement of mRNA abundances with digoxigenin-labeled riboprobes [20,21]. Rather, colorimetric ISH signal is believed to reflect only relative assessment of the amount of signal in the region and to measure linear quantitative changes in the transcript copy number. The tyramide amplification step [22] of non-radioactive ISH, necessary for detecting low transcript concentrations and signal identification, makes calculating the exact number of mRNA molecules per cell untenable mainly due to signal amplification, variations in probe permeability into the cell, changes in cell volume, and probe accessibility to mRNA. Nevertheless, qualitative comparison between radioactive and non-isotopic ISH shows very high degrees of concordance [2], and there have been several successful approaches toward relative quantification and semi-quantification in the sense of relative grading of signal intensity [23-26].

Gene expression microarrays remain a dominant technology in profiling genomic scale expression. Oligonucleotide arrays such as the widely popular commercial Affymetrix GeneChip™ [27] platform have the added benefit of reproducibility and convenience in large-scale experimentation. Significant variability in microarray data generation and subsequent errors in analysis arise from many sources such as use of non-identical samples on different platforms, variable protocols, insufficient replicates, lack of preprocessing standardization, and ambiguous post processing analysis. These results have been noted and confirmed in an expanding literature [28-38]. The results of cross-platform comparisons have also been mixed and continue to be debated [28]. Additionally, whereas several studies have raised concerns about the reliability of utilizing microarray data across and within platforms [30,33,39], more recent comparisons designed with careful control for probe homology and near identical implementations have reported higher concordance in differentially expressed genes [28]. Efforts to mandate standards and documentation help to improve microarray data reproducibility, for example, through Minimum Information About a Microarray Experiment (MIAME) [40] and the Microarray Quality Control (MAQC) project [41].

It is worth considering the technical and practical challenges in comparing microarray data with large-scale colorimetric ISH data. There are three main issues in comparing these data modalities. First, the probe sequences differ in length and/or localization along the target transcript. Oligonucleotide arrays use a series of short highly specific sequences to identify transcripts (Affymetrix, 20-25 nucleotides), whereas digoxigenin-based ISH typically uses a much longer (400-1,000 nucleotides) riboprobe; therefore, these longer probes likely represent 'pan-splice variant' probes, while the shorter probes may target specific splice variants that are differentially regulated. Second, there are significant differences in the measurable dynamic range. Affymetrix microarrays have ranges nearly two orders of magnitude greater than colorimetric ISH signal, with typically a ten-fold gain in the highest expressing decile. Colorimetric ISH, in contrast, has a compressed upper range, largely due to signal saturation induced by tyramide amplification [22]. While normalizing transformations are possible [42] and the reaction rate in ISH can be adjusted to reduce high end saturation [43], this latter action may be at odds with standardized platform comparison, and essential incompatibilities in signal representation between platforms remain. Even quantitative RT-PCR, commonly accepted as a gold standard for relative gene expression, and with an impressive dynamic range of order 10^7^, has been shown to have biases for genes with lower or variable expression rates [44]. Finally, normalization and accurate mapping of ISH data present unique challenges, and are essential to allow cross-gene and cross-structure analysis. Summarizing ISH expression numerically necessitates some form of spatial normalization or mapping to ensure that like regions are compared [17].

With the completion of the first genome-scale ISH project, the ABA, and with the initiation of other similar large scale data sets, it is essential to develop methods for genome-scale analysis of these data and to understand their relationship to other genome-scale expression profiling platforms [45]. In the current study, a metric for relatively quantifying colorimetric ISH signal suitable for cross-platform comparison is introduced. The method is novel in that it attempts to define an expression level analogous to microarray expression level by using cellular resolution image segmentation corroborated by tissue optical density. The large-scale ISH mapping environment of the ABA [2,17] and associated data validation studies are described, followed by comparison to two publicly available microarray data sets (Teragenomics [5] and Genomics Institute of the Novartis Research Foundation (GNF) [46]) for six brain structures: the striatum, cortex, cerebellum, hippocampus, olfactory bulb, and hypothalamus. Several types of correlation results are presented, including categorical and numerical correlation using a structural ratio approach designed to minimize cross-platform variability. The final section demonstrates advantages of using ISH for identifying distinct patterns of sub-structural and cell type-specific expression at a given microarray level.

## Results

### Relative quantification of colorimetric ISH data

**Figure 1:**
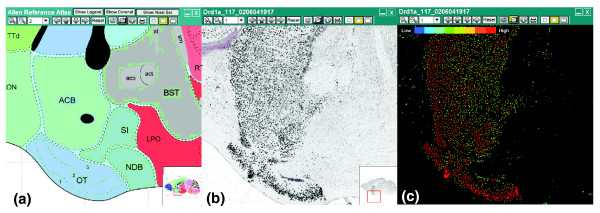
Signal segmentation of ISH data. **(a) **Sagittal plane annotated drawing from Allen Reference Atlas containing the olfactory tubercle (OT), nucleus accumbens (ACB) and caudoputamen (above ACB). **(b) **Plane-matched colorimetric ISH data for the gene *Drd1a*. **(c) **Corresponding expression segmentation mask for the ISH image shown in (b). High expression is indicated by red and low expression by blue.

Gene expression signal in colorimetric ABA ISH data is classified using an automated image segmentation algorithm (described in detail in [2,17]) that identifies contiguous groups of pixels corresponding to higher visual concentrations of BCIP/NBT (5-bromo,4-chloro,3-indolylphosphate/nitroblue tetrazolium), a precipitate deposited at riboprobe binding sites in the cell. Working from a 10× magnification (1.05 μm/pixel) high-resolution ISH image (Figure 1b), the algorithm segments and labels putative expressing cells using a statistical classifier [47] that exploits image intensity distribution and cell shape (morphology) characteristics [17]. Typical non-radioactive ISH images can have rather high levels of background intensity contaminated with non-specific hybridization products that resemble low-level expressing cells. The ABA ISH expression detection algorithm is designed to focus on achieving a high recognition probability for medium/high-level expressing cells while maintaining high specificity for rejecting non-specific labeling and artifacts among low expressing genes [17]. The algorithm output is an expression segmentation heat mask with measurable characteristics (Figure 1c). High concordance between automated and manual segmentation using these methods has been previously demonstrated [17].

The traditional approach to quantifying radioactive isotopic ISH has been to use optical density [11,48] of the underlying tissue section. If the probe specific binding activity is known, then the number of probe molecules bound to a certain tissue area can be calculated [15,18]. In both radioactive and non-radioactive ISH, most rigorous measures of quantification have employed optical density of the image area relative to background as a measure of signal intensity, and in each case increased optical density correlates with either the number of mRNA molecules (radioactive) or increased mRNA content (non-radioactive) [15,49].

In order to correlate gene expression across-platforms and between distinct brain regions with different underlying cell densities, a new variant of integrated optical density that is normalized to the cell density in each region was generated. For any given cell, ISH signal is a function of both intensity and fractional area occupied within the cell. To generalize this to a region R, expression level *L *is defined as:

$$L_{R}=\frac{a_{R}}{a_{\max}}\;{\bar{I}}_{R}$$

that is, the product of $\bar{I}$ the average pixel intensity for all expressing cells in *R*, times the relative fractional area occupied by expressing cells in *R*. This latter ratio is obtained by dividing the area of expressing cells *a*_*R *_in *R *by the effective maximum possible expression area *a*_*max *_in *R*, computed by applying the detection algorithm to a set of ubiquitously expressed genes [2]. The ubiquitously expressed genes (*H2-T3*, *Ube2c*, *0610008A10Rik*, and *1810037K07Rik*), genes expressed in nearly every cell, were identified through manual curation and possess the maximum observable expression throughout the brain. Expression level *L *is, therefore, a fraction of the average 8-bit integer pixel intensity and ranges from [0,255], and all quantities are computed from masks similar to that shown in Figure 1c. Expression level as defined is, therefore, a cell density normalized ISH analog to microarray level that models transcript copy number in a given region *R*, assuming both average intensity and fractional area of expressing cells scale with transcript count.

The correlation between the expression level *L *calculated by the ABA expression detection algorithm and integrated optical density [48] was tested. Measurements from expressing cells were made from the caudoputamen from 30 ISH images for six genes, five sections per gene (Table S1 in Additional data file 1). Figure S1 in Additional data file 1 shows a high correlation (R = 0.99), as expected for a fixed region, between expression level and optical density measured at a calibrated microscope and indicates that ISH expression level scales with relative mRNA signal and transcript count to the extent that optical density is a realistic measurement of that signal.

**Figure 2:**
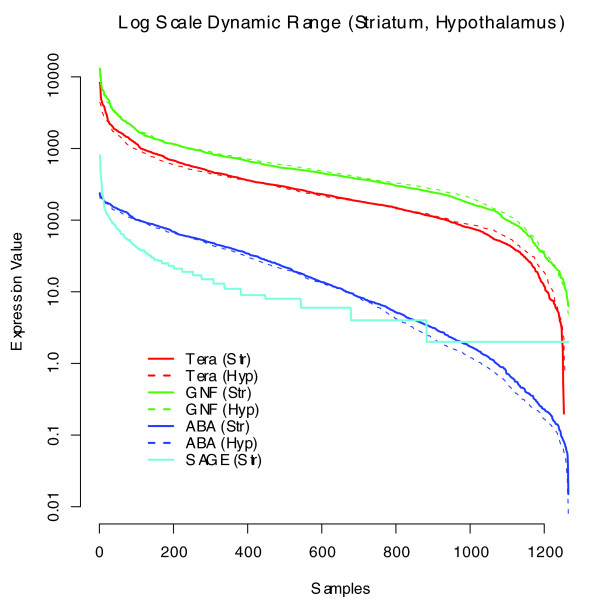
Cross-platform comparison of global dynamic range for microarray, ISH, and SAGE. Dynamic range of signal intensities in the striatum (Str; solid lines) and hypothalamus (Hyp; dashed lines) observed in GNF (green lines), Teragenomics (Tera; red lines), ABA (blue lines), and SAGE (aqua line) data sets (striatum only). The data are plotted on a log scale for 1,270 of the highest expression values. Genes on each curve are sorted independently so that only the relative range of values is preserved. The compressed dynamic range at the highest levels in ISH quantification compared to the microarray and SAGE platforms is notable.

The dynamic range of the method was then investigated. This range remains limited compared to other platform expression metrics, with truncation in the higher ranges due to signal saturation. Figure 2 plots on log scale 1,270 of the highest sorted expression values for Teragenomics, GNF, and ABA common genes in the striatum and hypothalamus. (A SAGE library for the striatum is included for reference [50]). Genes on each curve are sorted independently so that only the relative range of values is preserved. The profiles are nearly identical across structures and show that the dynamic range of the highest 15% of values is comparatively flat for colorimetric ISH while decreasing by a factor of 10 for microarray data and by at least a factor of 20 for SAGE. While some rescaling of the ISH dynamic range is possible to some extent, this essentially would have to be implemented on a gene by gene basis [51].

A particular advantage of colorimetric ISH is that other statistics, such as the proportion of labeled expressing cells, can be calculated. Expression density *D *in a region *R *is defined as:

$$D_{R}=\frac{n_{R}}{n_{\max}}$$

where *n*_*R *_is the number of detected expressing cells in *R *and *n*_*max *_is the approximate theoretical maximum computed as above. If all cells were spherical with identical volumes and ISH signal uniformly filled those cells, then expression level would scale linearly with density. However, in real tissue the relationship between level and density can be complex [2], as illustrated in the final section. It is also possible to develop quantitative measures of ISH signal uniformity across a structure using methods of spatial statistics, such as the k-estimator of Ripley [52].

### ABA ISH platform validation

To perform genomic scale signal mapping, quantification, and replicate analysis in the ABA requires utilizing the spatial mapping platform previously described in [2,17]. Briefly, in this pipeline, image series are white balanced and cropped, then reconstructed and registered to a three-dimensional informatics reference atlas for the C57BL/6J mouse brain [53]. Expression is quantified according to level and density metrics on a per section basis, and the results for three-dimensional anatomic regions can be compiled using the registration reconstruction and the virtual reference atlas. Details can be found in [17] and on the web site [3].

**Figure 3:**
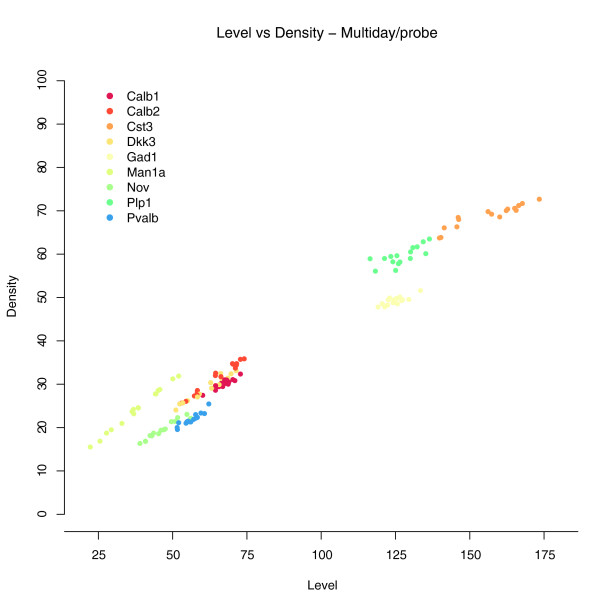
ABA ISH data repeatability. A scatter plot is shown for the average level (x-axis) and density (y-axis) measurements over a multi-day and multi-probe data set. For each of 9 genes (*Calb1*, *Calb2*, *Cst3*, *Dkk3*, *Gad1*, *Man1a*, *Plp1*, *Pvalb*, and *Nov*), 16 repeated measurements of expression level [0,255] and density [0,100] for an entire brain hemisphere are plotted. Probes were independently generated by *in vitro *transcription four times on four different days. Each independently synthesized probe was hybridized to brain tissue sections over a span of four days.

To examine the fidelity of this high-throughput platform to produce consistent quantification of regional ISH signal, a set of experiments were performed to assess variation in hapten incorporation into riboprobes, day-to-day variability associated with reagent preparation, and accuracy of mapping signal to brain anatomy. To assess the first two factors, an experiment was designed to compare ISH quantification results for nine genes (*Calb1*, *Calb2*, *Cst3*, *Dkk3*, *Gad1*, *Man1a*, *Plp1*, *Pvalb*, and *Nov*) processed independently on four different days. On each day, four independent riboprobes were also synthesized for each gene by *in vitro *transcription. The data generated for each gene consisted of 16 complete sagittal series (20 tissue sections per riboprobe) through a single brain hemisphere. Figure 3 shows a scatter plot of the computed level and density measurements at the whole brain structure level, computed hemisphere wide for 9 genes × 4 riboprobes × 4 days. Clustering of these data reveals the fidelity of this approach, in that the nearest neighbor in (level, density) space to any given gene is a replicate of the same gene in 81.8% of all cases, or a second nearest neighbor in 90.3% of cases. These results demonstrate the consistency of the ABA ISH platform day to day, treatment to treatment, and sample to sample and the reproducibility of the values level and density at the brain-wide level. It is notable that level and density values even at the level of the entire brain essentially uniquely identify this set of genes.

**Figure 4:**
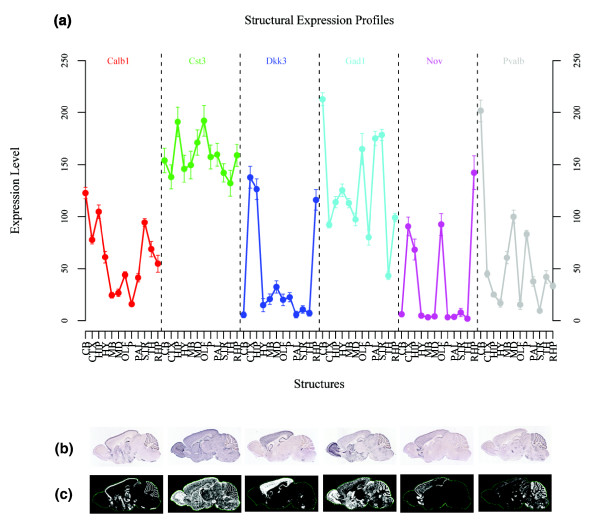
Structural profile plot for expression level for six control genes, *Calb1*, *Cst3*, *Dkk3*, *Gad1*, *Nov*, and *Pvalb*. **(a) **Expression levels with error bars for 4 days × 4 probe measurements after three-dimensional anatomic mapping. **(b) **Original ISH images at mid-sagittal plane. **(c) **Segmentation expression mask of corresponding ISH sections (b) used as the basis for quantification of expression level and density. CB, cerebellum; CTX, cortex; HIP, hippocampus; HY, hypothalamus; MB, midbrain; MD, medulla; OLF, olfactory bulb; P, pons; PAL, pallidum; STR, striatum; TH, thalamus; RHP, retrohippocampal region.

To examine the fidelity of the automated structural registration with respect to signal measurement, expression levels in different brain regions for the same data set described above were compared. As shown in Figure 4a, a unique reproducible profile emerges for each gene's expression pattern, highly consistent with the original ISH shown in Figure 4b and corresponding expression segmentation map in Figure 4c. The mean variation in signal across genes from laboratory through informatics processing is only 5.09%, with day-to-day variability 0.26% greater than probe synthesis variability (Additional data file 2). These control experiments establish key criteria for making global cross-gene and cross-structure comparisons, namely reproducibility of high throughput, semi-automated ISH data generation, mapping and quantification.

It should be noted that these control experiments are somewhat idealized. First, ABA data were generated over approximately a three-year period, with consequently larger process variation than the experiment described above. Second, the Allen Reference Atlas model used for structural mapping was produced in the coronal plane, while ABA ISH data for about 78% of genes are available only in the sagittal plane. Cross-axis registration is somewhat less reliable than same-plane registration. To investigate these effects further, 2,263 genes with more than one replicate were selected: either genes with two image series in the sagittal plane (1,828) or genes with one replicate in the sagittal plane and the other in the coronal plane (425). The correlations of ABA ISH expression level between the replicates retain high repeatability (ρ > 0.8) for all six structures with the correlation for the sagittal and coronal plane data ranging from 0.790 to 0.873 (lowest correlation in hippocampus and highest correlation in striatum). The correlation between the sagittal data sets ranged from 0.835 to 0.950, with the lowest and highest correlation in the same respective structures. These results are based on applying the automated spatial mapping process and the correlation results should, therefore, be understood as lower bounds for reproducibility of the replicate signals.

### GNF and Teragenomics microarray data sets

Several studies to date have investigated the relative internal consistency of microarray platforms under a variety of experimental conditions [28-33,54-56], and high degrees of within platform consistency have been reported for Affymetrix oligonucleotide microarrays [28,39,41]. In this study, several publicly available Affymetrix-based microarray data sets were chosen for analysis. The selected data sets profile different brain regions in mouse strains and ages similar to those used for the ABA (C57BL/6J, P56), and had replicate data. Both Teragenomics [57], using the Affymetrix U74Av2 platform, and GNF [58], using a custom Affymetrix array design, present two replicates for each of the six brain structures examined (with the exception of cortex, for which only one replicate was available from GNF) (see Materials and methods; Table 1, and Additional data files 3 and 4). The condensation algorithm used for GNF data was MAS5 and a close variant was developed by Teragenomics (Matthew Zapala, personal communication). Correlation between replicates was calculated to examine the variability within each microarray platform. To avoid probe to gene mapping issues, the comparison data set was limited to genes that had a one-to-one mapping between probe and gene (see Materials and methods; Table 2).

**Table 1:** Gene expression platforms and data used in the cross-platform comparison

Technology	Platform	Unique probe sequences	Unique probe sequences with Entrez IDs	Mouse strain	Mouse age (weeks)	Mouse sex
Microarray	**GNF **[58], Affymetrix GNF1M	31,777	26,982	C57BL/6J	10-12	M and F
	**Teragenomics **[57], Affymetrix MG-U74Av2	12,488	11,779	C57BL/6J	8	M
ISH	**ABA **[3]	25,739	21,677	C57BL/6J	8	M

The table shows the technology, followed by the source of the publicly available data (platform), number of probes with unique sequences (unique probe sequences), number of probes/sequences that have Entrez IDs (unique probe sequences with Entrez IDs), and the biological samples used to generate the expression data (mouse strain, age, and sex). Replicates for GNF consist of GNF1 and GNF2 and are identified by the column names of the downloadable data file GNF1M, MGJZ030207007A and MGJZ030207007B, respectively. Teragenomics replicates (TERA1 and TERA2) are specified by GEO IDs and are listed in the Materials and methods.

**Table 2:** Gene selection for ISH and microarray expression comparison

	All genes	Genes with Entrez ID	One-to-one probe/gene mapping	Consistent present/absent call	Present and consistent
**ISH (ABA)**					
Striatum	25,739	21,677	17,066	16,877	8,919
Cortex	25,749	21,683	17,077	16,848	11,358
Cerebellum	25,754	21,690	17,075	16,850	11,393
Hippocampus	25,766	21,691	17,074	16,897	11,465
Olfactory bulb	25,748	21,686	17,078	16,853	11,979
Hypothalamus	25,715	21,652	16,593	16,465	7,188
**Microarray (Teragenomics)**					
Striatum	12,488	11,779	7,062	6,026	3,499
Cortex	12,488	11,779	7,062	5,958	3,488
Cerebellum	12,488	11,779	7,062	6,066	3,470
Hippocampus	12,488	11,779	7,062	5,997	3,363
Olfactory bulb	12,488	11,779	7,062	5,987	3,593
Hypothalamus	12,488	11,779	7,062	6,099	4,000
**Microarray (GNF)**					
Striatum	31,770	26,982	13,582	11,713	3,438
Cortex	31,770	26,982	13,582	12,845	4,746
Cerebellum	31,770	26,982	13,582	11,773	4,121
Hippocampus	31,770	26,982	13,582	11,777	3,837
Olfactory bulb	31,770	26,982	13,582	11,885	4,123
Hypothalamus	31,770	26,982	13,582	11,869	4,341

Tabulated numbers show the number of genes satisfying the given criteria. Consistent present/absent reduces gene counts from one-to-one probe/gene mapping for genes with inconsistent replicates or genes with ambiguous expression call. (The ABA data contain a limited number of sagittal replicates: striatum 698, cortex 700, cerebellum 697, hippocampus 699, olfactory bulb 700, and hypothalamus 666.) The final column indicates those genes that are labeled as having present expression by present/absent calling algorithms for each platform minus those genes without consistent calls for those data available in replicate.

**Figure 5:**
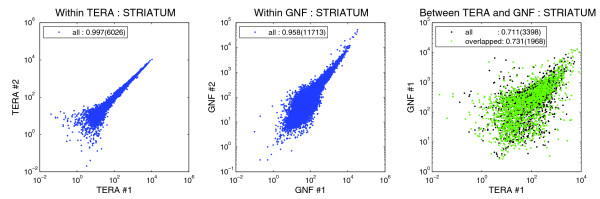
Intra- and cross-platform comparison between GNF and Teragenomics data sets for the striatum. Scatter plots showing correlation of expression levels between replicates in Teragenomics (TERA; left panel), replicates in GNF (center panel), and cross-platform for Teragenomics and GNF (right panel). Correlations and gene numbers are from Table 3. Scatter plots for the other five structures are shown in Figure S2 in Additional data file 1.

It is well known that many factors contribute to variation between microarray experiments even when using the same platform, including probe preparation and experimental protocols [28,33,42]. Table 3 shows a very strong internal correlation between replicates (average ρ > 0.98 for Teragenomics and ρ > 0.96 for GNF) throughout all six structures. The correlation decreases to an average of 0.724 when comparison is made between the two Affymetrix platforms for a given brain structure. Excluding genes with no overlap between probe sequences from each platform (42%), the correlation shows slight but not statistically significant improvement at the α = 0.05 level in all six structures (Table 3). Scatter plots for correlation in the striatum are shown in Figure 5. All six structures have similar results (Figure S2 in Additional data file 1).

**Table 3:** Teragenomics and GNF microarray platforms show strong intra-platform repeatability

Structure	TERA1, TERA2	GNF1, GNF2	GNF1, TERA1	GNF1*, TERA1*
Striatum	0.997 (6,026)	0.958 (11,713)	0.711 (3,398)	0.731 (1,968)
Cortex	0.985 (5,958)	NA	0.728 (3,693)	0.757 (2,129)
Cerebellum	0.995 (6,066)	0.977 (11,773)	0.731 (3,490)	0.756 (2,035)
Hippocampus	0.992 (5,997)	0.975 (11,777)	0.732 (3,410)	0.755 (1,985)
Olfactory bulb	0.994 (5,987)	0.987 (11,885)	0.727 (3,437)	0.749 (1,992)
Hypothalamus	0.980 (6,099)	0.994 (11,869)	0.716 (3,479)	0.738 (2,017)

Pearson correlation of expression levels is shown followed by the number of genes. Only one replicate from Teragenomics (TERA) and GNF (GNF1) is used in the inter-platform comparison (defined in Materials and methods). *Results are based on genes with positive overlapping probe sequences determined from NCBI BLAST alignments for Teragenomics and GNF probe sequences against the target gene sequence. NA indicates that there is only one GNF cortex data set.

### Cross-platform correlation of gene expression

**Figure 6:**
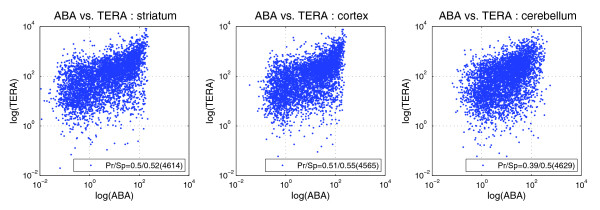
Scatter plots for ISH ABA expression level versus Teragenomics (TERA) level for striatum, cortex, and cerebellum. Pearson (Pr) and Spearman (Sp) correlations are given in the figure with gene numbers shown in parentheses. Scatter plots for the other structures and for ABA versus GNF are shown in Figure S3 in Additional data file 1.

Expression level and density are computed in the ABA pipeline for each anatomic structure (Additional data file 5) and can be correlated with other data modalities. The standard correlation for colorimetric ISH expression level (as defined above) versus microarray level was first considered. A single replicate is used in computing correlation as the internal consistency of both GNF and Teragenomics is high, and approximately 80% of ABA data is single replicate. Representative scatter plots for ABA versus Teragenomics for expression level are shown in Figure 6 (data for additional structures are in Figure S3 in Additional data file 1) and values are presented in Table 4 for ABA versus GNF and ABA versus Teragenomics with Pearson and Spearman correlations. The table shows weaker agreement of ISH data with either of the microarray data sets than the cross-microarray GNF versus Teragenomics correlation. While the difference is obviously significant, which of GNF or Teragenomics is closer to ABA can be tested by applying a Fisher r-to-z transformation [59] for observed difference in correlation. An asterisk in Table 4 indicates that the ISH Pearson correlations in the first two columns are distinct at significance level α = 0.05, and the only structures whose correlations are significantly different are cerebellum and olfactory bulb. In terms of Pearson correlation, the weighted average ABA-GNF correlation (0.479) is quite similar to the ABA-Teragenomics correlation (0.471), while the average ABA-GNF Spearman correlation (0.401) is somewhat weaker than the corresponding ABA-Teragenomics value (0.526).

**Table 4:** Comparison of correlation coefficients for Pearson/Spearman correlations between ABA, GNF, and Teragenomics data sets

Structure	ABA, GNF1	ABA, TERA1	GNF1, TERA1
Striatum	0.490/0.373 (8,669)	0.503/0.520 (4,614)	0.711/0.618 (3,398)
Cortex	0.483/0.418 (9,502)	0.506/0.549 (4,568)	0.728/0.647 (3,693)
Cerebellum	0.417/0.388 (8,726)*	0.385/0.496 (4,631)*	0.731/0.661 (3,490)
Hippocampus	0.506/0.427 (8,751)	0.505/0.578 (4,585)	0.732/0.644 (3,410)
Olfactory bulb	0.464/0.403 (8,811)*	0.436/0.515 (4,582)*	0.727/0.656 (3,437)
Hypothalamus	0.518/0.396 (8,571)	0.492/0.501 (4,566)	0.716/0.658 (3,479)

Each correlation (Pearson/Spearman) is shown followed by the number of genes. *Pearson correlation in the first two columns for cerebellum and olfactory bulb differ at a significance level α = 0.05.

**Figure 7:**
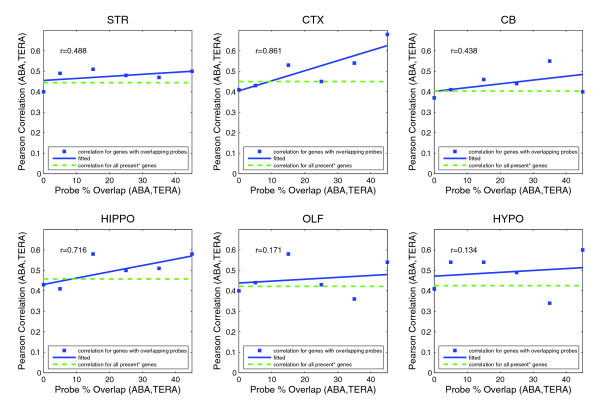
Pearson correlation between ABA and Teragenomics (TERA) data sets as a function of probe homology. Probe overlap ranges from 0 to 50%. Cortex (CTX) and hippocampus (HIPPO) exhibit the strongest positive improvement of correlation with probe homology, yet the trend is positive for the other four structures (STR, striatum; CB, cerebellum; OLF, olfactory bulb; HYPO, hypothalamus).

To investigate the effect of increasing probe homology on platform correlation, Pearson correlation between expression levels as a function of increasing probe base-pair agreement after BLAST alignment [60] of probe sequences to the target gene was computed. Figure 7 shows ABA-Teragenomics correlation as probe overlap ranges from disjoint to 50%. A regression fit to the data with R^2 ^shown in Figure 7 illustrates strong increasing correlation with probe homology for cortex (R^2 ^= 0.861) and hippocampus (R^2 ^= 0.716) and weaker, although positive, improvement for other structures. Thus, the results are expectedly strengthened by considering more homologous targets.

To more fully account for significant data modality and platform differences, a technique originally proposed by van Ruissen *et al*. [30] was modified in which microarray and SAGE cross-platform comparisons are made based on correlation of ratios of expression values for transcripts. Conceptually, cross-platform variability should be accounted for by considering expression ratios rather than correlation of absolute levels. This technique was generalized to enable structural comparisons as follows: for each of six structures S, each data point input to the correlation is the log ratio of gene expression level of S to any of five other structures. (Using the cortex as an example, cortex/striatum, cortex/olfactory bulb, cortex/hypothalamus, cortex/hippocampus, and cortex/cerebellum). Similar ratios were obtained for all structures, provided the denominator structure exhibits positive expression density D and provided the gene is called 'present' in at least one of the two structures forming the ratio. In addition to reflecting the notion that the ratio of transcript abundance should be independent of inherent platform differences (particularly probe sequence differences), the ratio test is potentially a better comparison of relative abundance and is more consistent with colorimetric ISH expression level as a relative or qualitative measure.

**Figure 8:**
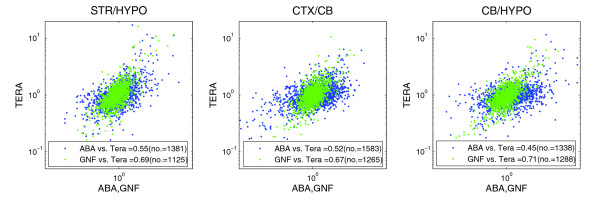
Structure level ratio scatter plots showing striatum (STR)/hypothalamus (HYPO), cortex (CTX)/cerebellum (CB), and CB/HYPO for ABA versus Teragenomics (TERA) and GNF versus TERA data sets. Pearson (Pr) and Spearman (Sp) correlations are shown with gene counts in parentheses. Scatter plots for the remaining structures and for ABA versus GNF are shown in Figure S4a and S4b in Additional data file 1.

**Figure 9:**
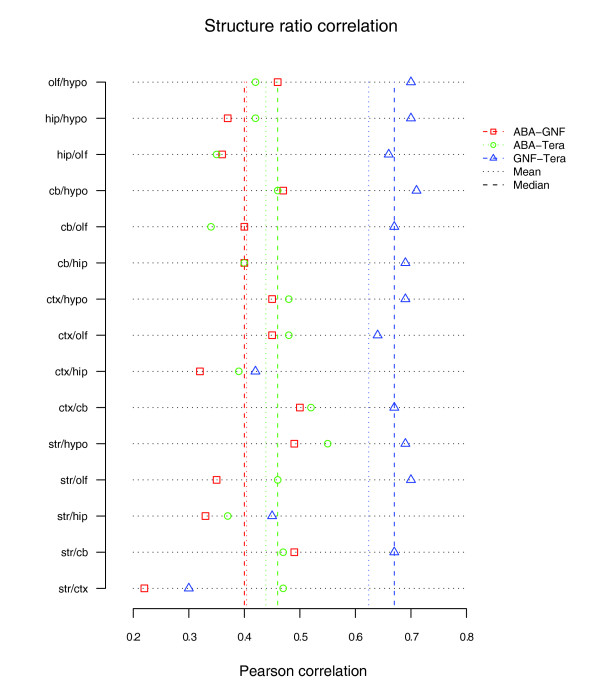
Structure ratio correlation summary for ABA, GNF, and Teragenomics. The mean values are shown as dashed lines. ABA-GNF values are in red, ABA-Teragenomics (Tera) values are in green, and GNF-Teragenomics values are in blue. ctx, cortex; hip, hippocampus; str, striatum; cb, cerebellum; olf, olfactory bulb; hypo, hypothalamus.

After normalizing the histogram of ratio values for each platform to a common scale following [30], Pearson and Spearman correlation was calculated over the log expression level ratios. Representative structure ratio scatter plots for ABA versus Teragenomics for expression level are shown in Figure 8 (the full set is available in Figure S4 in Additional data file 1). Figure 9 shows the summary results of this technique with mean (median) Pearson correlations: ABA-GNF, 0.404 (0.4); ABA-Teragenomics, 0.438 (0.46); and GNF-Teragenomics, 0.624 (0.67). On the whole, these results surprisingly do not illustrate a stronger correlation and are consistent overall with the standard Pearson approach (this result holds for Spearman as well (Figure S5 in Additional data file 1)) with a slightly weaker result for GNF-Teragenomics. The results can be considerably improved, however, by restricting the analysis to genes exhibiting higher expression fold changes. For each structure ratio pair *S*_1_/*S*_2_, those genes that exhibited at least a two-fold expression difference between the structures *S*_1_, *S*_2 _(for either structure) in the Teragenomics data set were chosen. For these gene subsets the correlation over all pairs improved to: ABA-Teragenomics mean, 0.73 (mean *N *= 159; range, 0.63 (hippocampus/olfactory bulb) to 0.79 (cerebellum/hippocampus)); ABA-GNF mean, 0.71 (mean *N *= 110; range, 0.51 (striatum/cortex) to 0.79 (striatum/cerebellum)); and GNF-Teragenomics mean, 0.84 (mean *N *= 97; range, 0.54 (striatum/cortex) to 0.91 (cerebellum/hypothalamus)). These results indicate that both ISH and microarrays do well at identifying genes with the highest relative expression differences between brain structures. The combined results also suggest that comparing colorimetric ISH with microarray data, as with any platform, may be more problematic for lower expressing genes.

### Categorical based platform comparison

To what extent do colorimetric ISH and microarray data yield reconcilable expression results? To address this question more fundamentally, correspondence at a binary present/absent level is considered. Utilizing binary present/absent calls for expression comparison has been noted as an oversimplification of true correlation due to class threshold effects [30], and alternative techniques have been developed [30,33,61]. However, this type of analysis may be appropriate where there are substantial differences in platform technology as is the case between colorimetric ISH and microarrays and to gain a benchmark.

A present/absent call for ABA ISH expression in each assayed structure was made by applying a threshold either to the expression level *L *or density *D*. With respect to density, when the threshold value for *D *is very low, numerous artifacts within ISH images may be detected as expression. On the other hand, if the threshold is too high, expression sensitivity is compromised. To determine an optimal threshold value a receiver operating characteristic (ROC) curve was drawn based on calling a gene expressed if its density attained a given value and by using ground truth determined from independent expert visual inspection. By examination of image series for 14,000 genes from the ABA, an optimal threshold for calling a gene expressed in a given structure based on expression density *D *was chosen by requiring that 1.0% of 8 × 8 pixel zones in that structure in the ISH image have expressing cells. A similar procedure can be used to threshold the ABA expression level, but thresholding on density *D *resulted in a closer call to visual inspection. This method yielded a sensitivity of 0.82 and specificity of 0.88 (Figure S6 in Additional data file 1).

Assessment of expression level for microarray data in terms of present/absent calls is generally made by algorithms counting transcript abundance [62]. For Teragenomics and GNF data, Affymetrix software [27] is used, and probe set detection p values are calculated relative to the intensity of all probes for a given gene (Additional data files 3 and 4). Since the Teragenomics and GNF data sets have strong repeatability (ρ > 0.98, and ρ > 0.96, respectively), only one replicate from each platform was used in this analysis.

Present/absent call results between ISH and the two microarray platforms for all structures are shown in Table 5. The present/absent call agreement ranges from 0.581 to 0.717 for ISH versus microarray, with the highest correlations between ISH and Teragenomics (0.687-0.717). The higher correlation for ISH and Teragenomics may be due to the fact the biological samples were more closely matched in age and sex, whereas the GNF data included male and female samples that were 2-4 weeks older. To test these results for significance, a two-tailed binomial proportions test [59] for observed difference in values was applied. An asterisk in Table 5 indicates that a given ABA correlation in either of the first two columns is statistically significant at level α = 0.05 when compared to the GNF replicate 1 (GNF1) versus Teragenomics replicate 1 (TERA1) proportion in the final column (see Materials and methods for the data sets comprising these replicates). Diagrams of the intersections of genes with present calls in the ABA, GNF, and Teragenomics platforms in six structures are displayed in Venn diagrams in Figure S7 in Additional data file 1.

**Table 5:** Present/absent call agreement among ISH and microarray platform thresholds with ISH thresholded by density

Structure	ABA versus GNF1	ABA versus TERA1	GNF1 versus TERA1
Striatum	0.639* (8,669)	0.717 (4,614)	0.715 (3,398)
Cortex	0.600* (9,502)	0.713* (4,568)	0.750 (3,693)
Cerebellum	0.589* (8,726)	0.687* (4,631)	0.773 (3,490)
Hippocampus	0.600* (8,751)	0.717* (4,585)	0.753 (3,410)
Olfactory bulb	0.581* (8,811)	0.709* (4,582)	0.759 (3,437)
Hypothalamus	0.678* (8,571)	0.689* (4,566)	0.737 (3,479)

The number of genes for each structure comparison is provided in parentheses. *Significance at α = 0.05 compared to GNF1 versus Teragenomics replicate 1 (TERA1) call agreement.

These results indicate that the ABA versus TERA1 binary correlation is slightly weaker than the GNF1 versus TERA1 binary correlation at 95% confidence, while it is as strong as in the striatum. The ABA versus GNF1 correlation is somewhat weaker than the GNF1 versus TERA1 binary correlation at much stronger confidence level. This result is consistent with a smaller scale manual comparison for embryonic ISH versus microarray presented in [14].

Variations on present/absent correlation are possible, including binary correlation of log ratios of expression levels for structures *S*_1_, *S*_2 _(that is, sgn log(*S*_1_/*S*_2_); Table S2 in Additional data file 1) and extensions to quartiles (low, low-medium, medium-high, and high; Figure S8 and Table S3 in Additional data file 1). These comparisons (Additional data file 6) show that these results are expectedly intermediate between binary and numerical correlation values.

### Spatial distribution and cell morphology: ISH expression level and density

**Figure 10:**
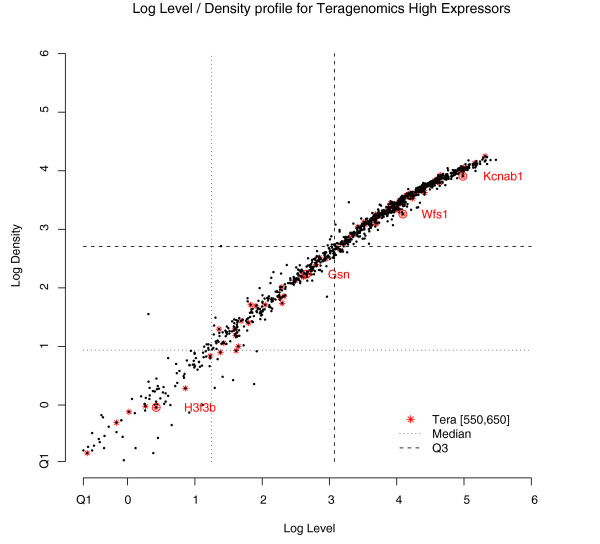
Log level/density ISH expression profile for Teragenomics high expressors. The plot shows 1,030 Teragenomics (Tera) high expressing genes in black with 84 genes in the limited microarray level range [550,650] marked with a red asterisk. The median (dotted line) and third quartiles (Q3, dashed line) are shown for both level and density. The first quartile (Q1) is shown at the axes origin. The genes labeled *H3f3b*, *Gsn*, *Wfs1*, and *Kcnab1 *are further discussed in the text and Figure 11.

The ability to examine high-resolution cellular morphology and spatial expression patterns is a notable advantage of colorimetric ISH in the analysis of gene expression. In addition to the visual component of viewing signal *in situ*, the relationship between quantifiable variables such as expression level and density (as defined above) yields more insight into relative and regional expression characteristics [2]. To explore this effect more thoroughly, the group of 1,030 genes defining the highest quartile of expression in the Teragenomics data set (shown in Figure 10) was considered, and a narrow microarray level range (550,650) between the median (480.7) and mean (766.7) level of this set, consisting of 84 genes, was selected. Figure 10 shows the ABA log expression level versus log density plotted in black for the full set of 1,030 genes with the 84 genes in the narrow microarray level range indicated by an asterisk. The dotted lines of Figure 10 are the median and third quartile values for level and density from the entire ABA data set.

From the distribution in Figure 10, the ABA level/density relationship for Teragenomics high expressors is essentially linear in log space (R^2 ^= 0.976), and all 1,030 genes are contained in the second to fourth quartile in both level and density (the first quartile is shown at the axes origin.). The quartile distribution of the 84 Teragenomics genes is (0, 7, 23, 54) for level and (0, 9, 22, 53) for density, and is consistent with the present/absent and categorical results presented earlier (see Additional data file 7 for expression values). Although there is some variation in the lower values of ABA level and density, the 84 Teragenomics high expressors are generally not among the outliers in Figure 10, with the possible exception of the gene *Wfs1*. In fact, small variations in the level/density profile of a gene can account for complexity in expression cell type and spatial pattern as shown below. To sample the variety and complexity that colorimetric ISH data may have within a limited microarray level range, four of the 84 genes were selected for consideration in detail.

**Figure 11:**
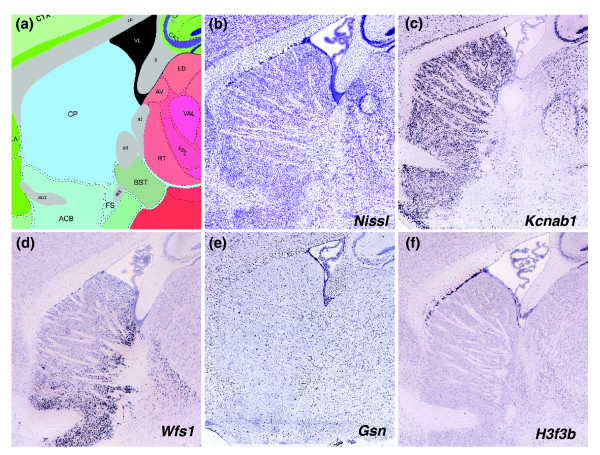
Four genes having Teragenomics expression level [550,650] showing complex regional and cell type expression in ISH. **(a,b) **Reference atlas annotation and corresponding Nissl for a section of the caudoputamen (CP) of the striatum. **(c) ***Kcnab1 *has widespread neuronal expression, while **(d) ***Wfs1 *is expressed in neurons but has a strong ventral gradient in the striatum with higher expression in the nucleus accumbens (ACB) than CP. **(e) ***Gsn *primarily labels the specific cell class oligodendrocytes and **(f) ***H3f3b *is expressed in the wall of the lateral ventricle towards the rostral migratory stream and is not expressed in the striatum *per se*.

Figure 11a shows the annotated image region from the caudoputamen of the striatum (Allen Reference Atlas [3]) and the corresponding Nissl stained section from the Allen Reference Atlas (Figure 11b). The remaining images are ISH images for plane matched sections of the caudoputamen for genes *Kcnab1*, *Wfs1*, *Gsn*, and *H3f3b*, all having similar microarray expression levels. The potassium voltage-gated channel, shaker-related subfamily, beta member 1 (*Kcnab1*; Figure 11c) is widely expressed in striatal neurons, and highly enriched in the striatum relative to other brain regions. Figure 11d shows expression of Wolfram syndrome 1 (*Wfs1*), a gene associated with Wolfram syndrome [63], a rare autosomal recessive neurodegenerative disorder characterized by simultaneous presentation of type I diabetes mellitus and optic atrophy in youth [64]. Unlike *Kcnab1*, *Wfs1 *is expressed in a subset of neurons in the caudal and ventral caudoputamen. The spatial expression pattern differences between *Kcnab1 *and *Wfs1 *are not apparent from their similar microarray expression levels but can be understood from Figure 10 as *Wfs1 *having a moderately reduced density for a given level compared with the more uniformly expressing *Kcnab1*.

Colorimetric ISH data also help to resolve the cell class by discriminating between non-neuronal and neuronal gene expression, and this cannot be distinguished by microarray levels alone. For example, Gelsolin (*Gsn*; Figure 11e), a Ca(2+) dependent actin regulatory protein [65], is expressed highly in oligodendrocytes [66], clearly visible by the high expression levels in white matter tracts surrounding the striatum and in scattered cells in the caudoputamen. A particularly compelling example of the value of cellular resolution is shown for the histone family member *H3f3b *(Figure 11f). Striatal detection of *H3f3b *by microarrays is apparently false and due to high levels of expression in the subventricular zone adjacent to the striatum that contains migrating cells in the rostral migratory stream. Even though *Hsfb3 *is reported positive for expression in the striatum by microarrays, the ISH data clearly show that *H3f3b *is not expressed in the striatum, but rather in the adjacent subventricular zone.

The ISH level and density profile in Figure 10 indicates that quantitative methods in colorimetric ISH may encode some variation in spatial pattern and cell type, even when restricted to a set of genes with a comparatively narrow range of high microarray expression level. The four genes shown in Figure 11c-f illustrate the range of expression characteristics (widespread, regional, cell class specific) that can be seen within a particular brain region with cellular resolution techniques. Expression ranges from widespread to highly restricted patterns and can include both neuronal and non-neuronal cell populations. While this heterogeneity can be resolved to some degree with methods for isolating much smaller tissue samples, such as microdissection [67] or voxellation [68], the resolution of colorimetric ISH for distinguishing intermingled cell populations remains a significant benefit of the technique.

## Discussion

Development of a technique for anatomic mapping and relative quantification of colorimetric ISH data has allowed the first large scale cross-platform comparison of this modality with publicly available microarray data. Colorimetric ISH and microarray data are found to agree well on higher expressing genes and in terms of presence/absence calls, although inherent cross-platform variability exists and is on the order of discrepancies observed between microarray and sequence-based profiling technology. For example, Liu *et al*. [69] found correlations in the range 0.45-0.51 when comparing multiple replicate microarray platforms to massively parallel signature sequencing (MPSS) methods such as SAGE, and two studies [30,39] found a range 0.42-0.54 for a similar comparison. Pearson correlation of expression level was also observed to be modestly improved by increasing probe homology, and significantly improved by removing lower expressing genes and considering ratio techniques.

Both microarrays and ABA ISH data show high internal reproducibility, while strict numerical correlations between ISH and microarray data sets are comparatively low, indicating that cross-platform discrepancies involve some differences in the fundamental underlying measurements. The correlations are substantially improved by moving to a binary present/absent correlation metric, suggesting that aspects of thresholding and differences in dynamic range are involved. Distinguishing signal from noise, and setting appropriate thresholds for signal detection, is difficult and a potential source of numerical discrepancy. A particular concern with quantifying ISH data is the prevalence of spurious tissue artifacts that could be misinterpreted for true signal. In designing the expression detection algorithm mask for the ABA ISH data [17], it was necessary to avoid these natural non-specific hybridization products, in the process reducing sensitivity on the lower end. On the other end of the scale, there is a compaction of the dynamic range due to signal saturation using colorimetric ISH produced with a single protocol for all genes (as for the ABA), illustrated in Figure 2. This is particularly the case for genes transcribed at very high copy number in relatively small populations of neurons, and is another potential source of discrepancy between the binary/categorical and numerical correlations. Although the best correlations were achieved with a binary present/absent correlation, there was good agreement for those genes exhibiting high cross-sample ratios, which is typically the highest value gene set.

It is difficult to account for all aspects of potential variability (sample preparation, probe design, spatial mapping accuracy, and so on) in comparing diverse platforms. Although the ABA ISH pipeline is highly standardized, more advanced informatics mapping tools, improved registration and signal detection might also improve the results further. As remarked in the introduction, however, there has been considerable debate on the efficacy of quantification measures for colorimetric ISH [15,18-21]. One of the key points emphasized in this work is that, while the absolute chemometric quantification for colorimetric ISH is unreliable, there is potentially great value in standardized relative signal measurement, particularly when complemented by accurate anatomic mapping. This combination enables a potentially wide range of rigorous studies, such as detection of expression level differences to within statistical significance, measurement of expression fold changes, and the study of other morphometric properties in gene expression only enabled by the spatial content of the signal. These advantages should justify the continued study and advancement of methods in colorimetric ISH quantification.

Concordance of gene expression between platforms has not been simplified by the addition of SAGE [1] to the repertoire [31,33,39,50], and one study indicates that Affymetrix, cDNA, and SAGE identified very different measures of coexpression for most gene pairs with very low correlation between platforms [33]. It would be useful to present a comparative analysis of ISH data with SAGE or one of its variants, such as cap analysis of gene expression (CAGE) [70]. However, at present there are no publicly available mouse brain SAGE data having replicates that closely match the ISH and microarray sample criteria. In the way of preliminary results, the correlation was calculated between two different SAGE libraries for the hypothalamus and striatum (Additional data file 8), which are available from the Cancer Genome Anatomy Project (CGAP) [71] (age P49) and the Mouse Gene Expression Atlas Project at the BC Cancer Agency (Mouse Atlas) [72] (age P84). These results are available in Table S4 in Additional data file 1 and indicate that the correlation between ISH and microarray platforms is approximately as close as that between SAGE and microarray data for the genes expressed in at least one of the two structures.

Measuring the transcript abundance (level) of a specific gene clearly depends heavily on the target probe for both microarray and ISH platforms. Most studies have found that both inter- and intra-platform comparisons are challenging due to annotation problems related to reconciling which genes are measured and detected by specific probes. In addition, possible one-to-many mappings between probes and genes hinder the ability to quantify expression, and efforts are underway toward RNA transcript-based annotation [73]. Earlier releases of Affymetrix GeneChip Murine Genome U74 reportedly used sequences from the public sequence databases that were ambiguous or on the wrong strand and, consequently, the oligonucleotides could not detect their target mRNAs [74]. This was resolved by Affymetrix prior to use in the Teragenomics data set (Affymetrix Statistical Algorithms Reference Guide, 2001 [75]) and was corrected in the GNF1M data set [76]. Whereas the Affymetrix platform uses probes of length approximately 25 bp, ABA riboprobes are much longer with a median length of 800 bp. Riboprobes are less prone to binding to non-targeted transcripts, but do have partial hybridization issues, particularly when families of related isoforms under alternative transcription are present [6]. As indicated above, these issues were avoided to the extent possible, although this study is based on limited replicates.

The intent of the final section of the paper is to illustrate the power gained from quantifying colorimetric ISH data over microarray data. ISH provides a cellular resolution generally unattainable by other techniques, and in addition to measures of expression level, important characteristics such as relative density and uniformity of expression across a structure can be quantified. Higher spatial resolution can be gained with microarray analysis by isolating smaller or more discrete tissues or cell populations, for example, using template based laser capture microdissection [77] or by techniques such as voxellation [78]. Several very promising methods for isolation of discrete cell populations for microarray analysis from transgenic animals have been recently described [79,80], although these approaches are currently limited by the availability of animals with labeled cell populations of interest. In practice, these two techniques are best seen as complementary for most research applications. Microarrays provide a robust, economical method to identify candidate genes that change in treatment versus control, mutant versus wild type, and so on, while ISH provides a deeper level of cellular specificity necessary to obtain a mechanistic understanding of altered gene expression in a complex cellular milieu. However, the availability of large-scale, baseline gene expression atlases such as the ABA provides a powerful resource to help select candidates from microarray experiments based on regionalized anatomical distribution or cellular specificity (for example, neuronal versus glial) of candidate genes in the unmanipulated condition.

Several large scale ISH projects for the rodent brain are proposed or underway. One such project is EurExpress [81], which is assessing gene expression patterns from approximately 20,000 genes obtained from embryonic day 14.5 (E14.5) mice by utilizing the ABA riboprobe templates. Another project [82] is an important source of data for the EurExpress project and is identifying murine expression patterns by colorimetric ISH starting at E10.5 on through adulthood, with an emphasis on gene expression in E14.5 [16]. The Embryo Gene Expression Patterns project [83] is generating colorimetric ISH data in three developmental stages, E10.5, E12.5 and E14.5. Other related projects that are producing gene expression data include the Gene Expression Nervous System Atlas (GENSAT) [84-87]. A larger scale radioisotopic ISH project is the Brain Gene Expression Map (BGEM), which identifies candidate genes for the GENSAT enhanced green fluorescent protein reporter transgenic mouse pipeline [85]. A shared characteristic of all ISH efforts is that some framework of anatomic mapping and quantification is necessary to enable both internal and cross-platform comparison, as well as to take full advantage of the significance of observed signal in the data. Much work remains to be done in this area and independent efforts are underway [88-91].

## Conclusion

New large scale implementations of colorimetric ISH are enabling high-throughput approaches such as the ABA. Despite the aforementioned challenges and difficulties of comparison, it is essential to reconcile this modality with existing standards. Numerous studies have illustrated the care in process and interpretation required to obtain scientifically meaningful results with microarray data, and these concerns are certainly not diminished with high-throughput ISH comparisons. Both microarray and colorimetric ISH are gene expression platforms with considerable variability, yet valuable content is obtainable from both. The additional morphological and potential cell type information gained through colorimetric ISH make it a rich source for gene expression information, and whereas conventional expression profiling captures only an expression level, ISH enables multiple at least semi-quantitative metrics that have been seen to distinguish more subtle characteristics. It is anticipated that new methods of analysis and comparison for better understanding of ISH and its relationship to existing standards will accompany the numerous emerging high-throughput ISH data sets.

## Materials and methods

### Data

Adult mouse brain gene expression data for C57Bl/6J based on Affymetrix MG-U74Av2 chip (Teragenomics [5,57], Gene Expression Omnibus (GEO) GSE3594) and Affymetrix GNF1M chip (Mouse GNF1M(MAS5-condensed; GeneAtlas [46,58], GEO GSE1133) were used for the microarray platform data. Both data sets have two replicates for six brain structures: striatum, cortex, cerebellum, hippocampus, olfactory bulb, and hypothalamus, except GNF in the cortex, which has one. TERA1 and TERA2 are replicates specified by GEO IDs. Data sets for TERA1 for the striatum, cortex, cerebellum, hippocampus, olfactory bulb, and hypothalamus are GSM82978, GSM82974, GSM82988, GSM82952, GSM82986, and GSM82953, respectively. Data sets for TERA2 for the striatum, cortex, cerebellum, hippocampus, olfactory bulb, and hypothalamus are GSM82979, GSM82975, GSM82989, GSM82951, GSM82987, and GSM82954, respectively. GNF1 and GNF2 are replicates identified by the column names of the downloadable data file GNF1M, MGJZ030207007A and MGJZ030207007B, respectively. For ISH, data from the ABA was gathered for these six brain structures following [2]. Microarray, SAGE and ISH data are provided in Additional data files 3-5 and 8.

### Preprocessing

For ABA ISH data, genes with corresponding Entrez IDs and having positive expression values in one of six structures were mapped in the pipeline described in [2,17], including pre-processing steps, image registration and signal segmentation. From each registered ISH image series the expression level and density statistics defined can be computed using the formulas from signal segmentation masks (Figure 1c) on a structure basis. The gene selection process and counts in the comparisons are shown in Table 2. For microarray data, probes that mapped to a unique Entrez ID and have one-to-one mapping between gene and probe ID were chosen. Both Teragenomics and GNF microarray data sets have two replicates. Replicates were used in all platforms when available in order to examine the consistency in present/absent calls, excluding those with inconsistent values. The final column of Table 2 indicates genes labeled as having present expression by present/absent calling algorithms for each respective platform minus those genes without consistent calls for those data available in replicate (except for the ABA, which has minimal replicates in the sagittal plane). For ABA present/absent calling, the density threshold values are 1.2582 (striatum), 1.2267 (cortex), 0.7946 (cerebellum), 0.3198 (hippocampus), 1.7140 (olfactory bulb), and 1.9723 (hypothalamus). Following preprocessing, each structure has a variable gene count for comparison (Figure S9 in Additional data file 1).

ImagePro software (MediaCybernetics, Silver Spring, MD, USA) was used for the manual annotation of the region of interest and optical density measurements. The statistical computing package R [92] was used for figure generation.

### Comparison metrics

#### Correlation

Pearson and Spearman correlations were calculated using Matlab (v7.2.0.232, The MathWorks, Inc., Natick, Massachusetts, USA). For microarray intraplatform repeatability, the expression values of the replicates were used. For interplatform comparison, the log ratio of gene expression values over pairs of structures was used to avoid platform differences as described in [30].

#### Present/absent call agreement

Each platform makes a call whether each gene is expressed ('present') or not ('absent'). These calls from two platforms were compared in terms of a 2 × 2 contingency table. Call agreement is defined as the percentage of total genes either simultaneously called present or absent by both platforms.

### Measuring probe sequence overlap

Probe sequences of Teragenomics, GNF, and ABA were downloaded from their respective websites and target gene sequences were obtained from NCBI [93]. Affymetrix MG-U74Av2 chip for Teragenomics and Affymetrix GNF1M chip for GNF both have 16 probes for each target gene. In each platform for each gene, the corresponding probe sequences were mapped against the target sequence using NCBI BLAST [60] and the aligned locations were recorded, measuring sequence overlap for aligned locations. The significance of the correlation difference before and after filtering out genes with no overlap was tested since the sample sizes changed significantly.

## Abbreviations

ABA, Allen Brain Atlas; E, embryonic day; GENSAT, Gene Expression Nervous System Atlas; GEO, Gene Expression Omnibus; GNF, Genomics Institute of the Novartis Research Foundation; ISH, *in situ *hybridization; SAGE, serial analysis of gene expression.

## Authors' contributions

C-KL, SMS, and MH performed the main analysis of the data. C-KL, SP, LN, and CL constructed the informatics image analysis pipeline. CLT, SF, and MM assisted with ISH issues and interpretation. CS offered microscopy help. AJ provided overall project guidance. EL and MH wrote the manuscript. All authors have read and approved the final manuscript.

## Additional data files

The following additional data are available with the online version of this paper. Additional data file 1 contains numerous additional figures and tables. Additional data file 2 is the multi-probe/day expression level and density data. Additional data file 3 is the Teragenomics microarray data. Additional data file 4 is the GNF microarray data. Additional data file 5 is the ABA expression level and density data. Additional data file 6 is the quartile grouping of ABA expression level and density with microarray expression data. Additional data file 7 shows ISH and microarray expression values for a limited microarray range. Additional data file 8 contains the SAGE expression data used in the analysis.

## Supplementary Material

Additional data file 1 Additional figures and tables. Click here for file

Additional data file 2 Multi-probe/day expression level and density data. Click here for file

Additional data file 3 Teragenomics microarray data. Click here for file

Additional data file 4 GNF microarray data. Click here for file

Additional data file 5 ABA expression level and density data. Click here for file

Additional data file 6 Quartile grouping of ABA expression level and density with microarray expression data. Click here for file

Additional data file 7 ISH and microarray expression values for a limited microarray range. Click here for file

Additional data file 8 SAGE expression data used in the analysis. Click here for file
