# Supplementary material for: Quantitative methods for genome-scale analysis of in situ hybridization and correlation with microarray data
Source: Genome Biol. 2008 Jan 30;9(1):R23. doi: 10.1186/gb-2008-9-1-r23 (PMC2395252; doi:10.1186/gb-2008-9-1-r23)
Supplement: Additional data file 1 — Additional figures and tables. [file gb-2008-9-1-r23-S1.doc]

**Additional data file 1**

**Table S1.** Optical density measurements of ISH expression.

**.**

| **Gene** | **Number of sections** | **OD** | **CC (x10000)** | **IOD (x10000)** |
| --- | --- | --- | --- | --- |
| *Arc* | 5 | 8.08E-02 | 7.508685648 | 6.49313 |
| *Egr1* | 5 | 2.10E-01 | 41.49989769 | 41.80385 |
| *Fos* | 5 | 4.22E-02 | 1.256577564 | 0.788153 |
| *Gal* | 5 | 3.27E-02 | 0.079199524 | 0.0516 |
| *Hspa5* | 5 | 1.30E-01 | 29.0135211 | 31.1071 |
| *Th* | 5 | 3.93E-02 | 1.381080639 | 1.216102 |

OD, standard optical density (using standard OD calibration in ImagePro Software, MediaCybernetics, Silver Spring, MD); CC= cell count per 100um^2^ area. Integrated optical density (IOD): Reports the average intensity/density of each object.  This value is expressed in terms of the current intensity/density mode and calibration. Five individual sections were measured for each of six genes.

**Figure S1.** Expression level L in the caudoputamen showing high correlation (R=0.99) with integrated optical density. Expression level and integrated optical density (IOD) are presented on the x-axis and y-axis, respectively, in log scale. IOD measurements for each gene are plotted with the same symbol showing consistency of expression level and IOD.

**Figure S2**. Microarray repeatability shown in terms of correlation of expression levels in five structures (cortex, cerebellum, hippocampus, olfactory bulb, and hypothalamus) between replicates in Teragenomics (TERA) (left column), replicates in GNF (center column), Teragenomics and GNF (right column). For TERA and GNF in the right column, overlapping data is shown in green and all data is shown in blue. For GNF, replicates are unavailable for the cortex.

**A**

**B**

**Figure S3**. (A) Scatter plots for ISH ABA expression level versus Teragenomics level for hippocampus, olfactory bulb, and hypothalamus. (B) Scatter plots for ISH ABA expression level versus GNF level for striatum, cortex, cerebellum, hippocampus, olfactory bulb, and hypothalamus. Pearson (Pr) and Spearman (Sp) correlations are provided along with gene numbers in parenthesis.

After normalizing the histogram of ratio values for each platform to a common scale following van Ruisssen *et al*., 2005, Pearson correlation is calculated over the log expression level ratios. The comparison ABA-Teragenomics is shown in Figure S4a while that for ABA-GNF is shown in Figure S4b.

**Figure S4(a).** Each scatter plot shows the log ratio of gene expression between two structures having positive expression density D. All six structures were paired and subjected to the log ratio. Comparison between ABA and Teragenomics (TERA) is plotted in blue and that between GNF and Teragenomics in green.

**Figure S4(b).** Each scatter plot shows the log ratio of gene expression between two structures having positive expression density D. All six structures were paired and subjected to the log ratio comparison. The data shown is between ABA and GNF.

**Figure S5**. Structure ratio. Spearman correlation summary for ABA, GNF, and Teragenomics. The mean values are shown as dashed lines. ABA-GNF values are in red, ABA-Teragenomics (Tera) values are in green, and GNF-Teragenomics values are in blue. Abbreviations: ctx, cortex; hip, hippocampus; str, striatum; cb, cerebellum; olf, olfactory bulb; hypo, hypothalamus.

**Figure S6**. ROC curve of ABA present/absent call shows good agreement with visual inspection of expression data. The current operating point is shown in the blue square (88% specificity, 83% sensitivity).

**Table S2.** Binary correlation of log ratios of gene expression between two structures.

| **Structure1/Structure2** | **ABA vs. Teragenomics** | **ABA vs. GNF** | **Teragenomics vs. GNF** |
| --- | --- | --- | --- |
| STR/CTX | 0.54 (2046) | 0.68 (1800) | 0.57 (1126) |
| STR/CB | 0.64 (1919) | 0.66 (1634) | 0.66 (1071) |
| STR/HIPPO | 0.54 (2005) | 0.61 (1720) | 0.60 (1080) |
| STR/OLF | 0.55 (2060) | 0.60 (1748) | 0.65 (1110) |
| STR/HYPO | 0.67 (1842) | 0.60 (1481) | 0.72 (1125) |
| CTX/CB | 0.62 (2111) | 0.62 (2210) | 0.67 (1265) |
| CTX/HIPPO | 0.59 (2216) | 0.53 (2270) | 0.59 (1218) |
| CTX/OLF | 0.63 (2242) | 0.62 (2371) | 0.65 (1281) |
| CTX/HYPO | 0.53 (1860) | 0.59 (1836) | 0.70 (1330) |
| CB/HIPPO | 0.59 (2080) | 0.57 (2026) | 0.68 (1153) |
| CB/OLF | 0.58 (2113) | 0.58 (2156) | 0.68 (1243) |
| CB/HYPO | 0.59 (1784) | 0.60 (1677) | 0.71 (1288) |
| HIPPO/OLF | 0.60 (2205) | 0.58 (2195) | 0.67 (1185) |
| HIPPO/HYPO | 0.51 (1829) | 0.49 (1670) | 0.71 (1213) |
| OLF/HYPO | 0.53 (1868) | 0.53 (1754) | 0.74 (1293) |

Two structures, S_1_and S_2_, having positive expression density D, sgn(log(S_1_/S_2_)), are measured in terms of the correct detection rate from a 2x2 contingency table of sgn(log(S_1_/S_2_)) in two platforms.

**Figure S7.** Diagrams of intersections of “present” genes in ABA, GNF, and Teragenomics (TERA) platform for comparison in striatum, cortex, cerebellum, hippocampus, olfactory bulb, and hypothalamus. The number of genes for each cross platform comparison in each structure differs and is tabulated for the genes called “present” in each platform.

**Figure S8**. Quartile call agreement between ISH and microarray platforms. Top two rows: ABA density vs. Teragenomics level. Bottom two rows: ABA level vs. Teragenomics level. The four quartile divisions are low (dark blue), low to medium (light blue), medium to high (orange-yellow), and high (dark red) for striatum (STR), cerebellum (CB), cortex (CTX), hippocampus (HIPPO), olfactory bulb (OLF), and hypothalamus (HYPO). Quartile threshold values for each structure in three platforms are shown in Table S3. The 4x4 contingency tables are available in Additional data file 6.

**Table S3**. Quartile threshold values for ABA, Teragenomics, and GNF.

|  | **Threshold** | **STR** | **CB** | **CTX** | **HIPPO** | **OLF** | **HYPO** |
| --- | --- | --- | --- | --- | --- | --- | --- |
| **ABA**  density | Low | 0.38 | 0.81 | 0.87 | 0.85 | 1.11 | 0.16 |
|  | Medium | 2.55 | 3.34 | 6.33 | 5.53 | 6.30 | 1.50 |
|  | High | 14.98 | 9.50 | 25.05 | 14.96 | 19.57 | 14.40 |
| **ABA**  level | Low | 0.51 | 1.98 | 1.23 | 1.42 | 2.09 | 0.46 |
|  | Medium | 3.47 | 8.98 | 8.16 | 10.40 | 10.54 | 3.11 |
|  | High | 21.63 | 32.44 | 42.27 | 42.11 | 38.96 | 22.82 |
| **TERA**  level | Low | 20.70 | 19.10 | 21.61 | 19.45 | 20.92 | 24.66 |
|  | Medium | 94.69 | 94.84 | 96.99 | 91.44 | 93.74 | 101.13 |
|  | High | 260.90 | 267.46 | 256.24 | 248.95 | 270.23 | 266.80 |
| **GNF**  level | Low | 75.15 | 85.33 | 84.13 | 69.66 | 79.50 | 84.06 |
|  | Medium | 232.82 | 240.87 | 239.28 | 219.29 | 245.25 | 247.55 |
|  | High | 494.75 | 552.49 | 532.14 | 527.06 | 519.70 | 546.58 |

For each platform, the threshold values for expression density (ABA) and level (ABA, Teragenomics (TERA) and GNF) are shown. Abbreviations: STR, striatum; CB, cerebellum; CTX, cortex; HIPPO, hippocampus; OLF, olfactory bulb; HYPO, hypothalamus.

**Table S4.** Present/absent call agreement among ISH, microarray, and SAGE for the striatum and hypothalamus.

| **Structures** | **ISH vs. SAGE** | **TERA1 vs. SAGE** | **GNF1 vs. SAGE** |
| --- | --- | --- | --- |
| Striatum (MouseAtlas) | 0.751 (5602) | 0.857 (2746) | 0.552 (3502) |
| Hypothalamus (CGAP) | 0.552 (5680) | 0.804 (2505) | 0.531 (3720) |
| Hypothalamus (MouseAtlas) | 0.653 (5966) | 0.887 (3014) | 0.653 (3894) |

These results indicate that the correlation between ISH and microarray platforms is approximately as close as that between SAGE and microarray data for genes expressed in at least one of the two structures.

**Figure S9.** Diagrams of ABA, GNF, and Teragenomics (TERA) platform gene count intersections for comparison in striatum, cortex, cerebellum, hippocampus, olfactory bulb, and hypothalamus. The number of genes for each cross platform comparison in each structure differs and is tabulated following preprocessing.
